# miR-208a in Cardiac Hypertrophy and Remodeling

**DOI:** 10.3389/fcvm.2021.773314

**Published:** 2021-12-09

**Authors:** Xing-Huai Huang, Jia-Lu Li, Xin-Yue Li, Shu-Xia Wang, Zhi-Han Jiao, Si-Qi Li, Jun Liu, Jian Ding

**Affiliations:** ^1^School of Life Science and Technology, Xi'an Jiaotong University, Xi'an, China; ^2^Department of Orthopaedics, Jiangsu Provincial Hospital of Traditional Chinese Medicine, Affiliated to Nanjing University of Chinese Traditional Medicine, Nanjing, China

**Keywords:** miR-208a, cardiomyopathy, miRNA, hypertrophy, Trbp, Sox6

## Abstract

Various stresses, including pressure overload and myocardial stretch, can trigger cardiac remodeling and result in heart diseases. The disorders are associated with high risk of morbidity and mortality and are among the major health problems in the world. MicroRNAs, a class of ~22nt-long small non-coding RNAs, have been found to participate in regulating heart development and function. One of them, miR-208a, a cardiac-specific microRNA, plays key role(s) in modulating gene expression in the heart, and is involved in a broad array of processes in cardiac pathogenesis. Genetic deletion or pharmacological inhibition of miR-208a in rodents attenuated stress-induced cardiac hypertrophy and remodeling. Transgenic expression of miR-208a in the heart was sufficient to cause hypertrophic growth of cardiomyocytes. miR-208a is also a key regulator of cardiac conduction system, either deletion or transgenic expression of miR-208a disturbed heart electrophysiology and could induce arrhythmias. In addition, miR-208a appeared to assist in regulating the expression of fast- and slow-twitch myofiber genes in the heart. Notably, this heart-specific miRNA could also modulate the “endocrine” function of cardiac muscle and govern the systemic energy homeostasis in the whole body. Despite of the critical roles, the underlying regulatory networks involving miR-208a are still elusive. Here, we summarize the progress made in understanding the function and mechanisms of this important miRNA in the heart, and propose several topics to be resolved as well as the hypothetical answers. We speculate that miR-208a may play diverse and even opposite roles by being involved in distinct molecular networks depending on the contexts. A deeper understanding of the precise mechanisms of its action under the conditions of cardiac homeostasis and diseases is needed. The clinical implications of miR-208a are also discussed.

## Introduction

The heart acts like a pump. It incessantly contracts to deliver oxygen and nutrient-rich blood throughout the body. In response to cardiac damage, pressure overload and a variety of other pathologic insults, the heart often undergoes complex molecular, cellular and interstitial changes, termed cardiac remodeling. The progression of cardiac remodeling eventually leads to cardiac dysfunction, which is the major threat to human health and has become one of the leading causes of death in world (1).

Hypertrophy is a common type of cardiac remodeling. It is the primary response of cardiac muscle to elevated workload or myocardial infarction (2). Cardiac hypertrophy is believed to be adaptive and have a “compensatory” role in the premise that it can diminish oxygen consumption, normalize the systolic wall stress, and improve ejection performance. However, long-term and chronic stress (ex. hypertension or valvular disease) can result in pathological remodeling, characterized by the increase in the size of cardiomyocytes, the abnormal enlargement and thickening of the heart muscle, cardiac dysfunction and fibrosis (3, 4). Multiple biological processes participate in modulating cardiac hypertrophy and remodeling. At the cellular level, hypertrophic growth of cardiomyocytes can be induced by numerous signal cues, including biomechanical stress, neurohumoral and endocrine hormones, involves MAPK, PI3K-AKT, Calcineurin-NFATc and other signal pathways (5), and is accompanied by enhanced protein synthesis, reorganization of the cytoskeleton, metabolic shift from oxidative phosphorylation to glycolysis and adult-to-fetal switch on expression program of myosin isoforms (3, 6–8).

A class of small noncoding RNAs, termed microRNAs (miRNAs), was discovered as key regulators of gene expression more than 2 decades ago (9–11). These small transcripts are composed of approximately 21–25 nucleotides, and exert their functions primarily through translational repression or messenger RNA degradation by base-pairing with the mRNA targets. MiRNAs are widely presented in many kinds of organisms (11, 12), and most of them, are well conserved during evolution (13, 14). Presently, more than 2000 miRNAs have been discovered in humans, and it is believed that these miRNAs can modulate approximately 1/3 of genes in the genome (15, 16). Because miRNAs frequently have only modest effects on the expression of individual genes, they are often conceptualized as “fine-tuners”. However, a single miRNA can target numerous mRNA transcripts, thus, the accumulative effects of coordinated modulation of multiple downstream mRNA transcripts can substantially influence the functional outcomes (17).

Extraordinary effort has been devoted to study cardiac hypertrophy and remodeling, yet, the underlying mechanisms remain elusive. Many clues to the regulatory events were derived from the identification and characterization of new factors involved in the processes. Numerous miRNAs, including miR-1, miR-133, miR-208a, miR-499 and miR-22, have been implicated in cardiac remodeling and pathogenesis, adding a new dimension to the regulatory networks of cardiomyopathy (18–22). Studies on miRNAs have uncovered previously unrecognized mechanisms and provided novel insights into cardiac remodeling. In addition, due to the relative ease of pharmacological manipulation, the identified cardiomyopathy-related miRNAs also hold great potential as promising targets for therapeutic intervention. Here, we focus on a cardiac muscle-specific miRNA, miR-208a (miR-208), which plays key roles in regulating heart function and appears to be master organizer of cardiac remodeling to pathogenic stress. We summarize the findings on its regulatory effects and mechanisms, and propose several intriguing topics, which need to be resolved in the future. The clinical potential of miR-208a as a diagnostic biomarker and a therapeutic target is also discussed.

## Expression of miR-208a and ITS Regulation

**Figure 1:**
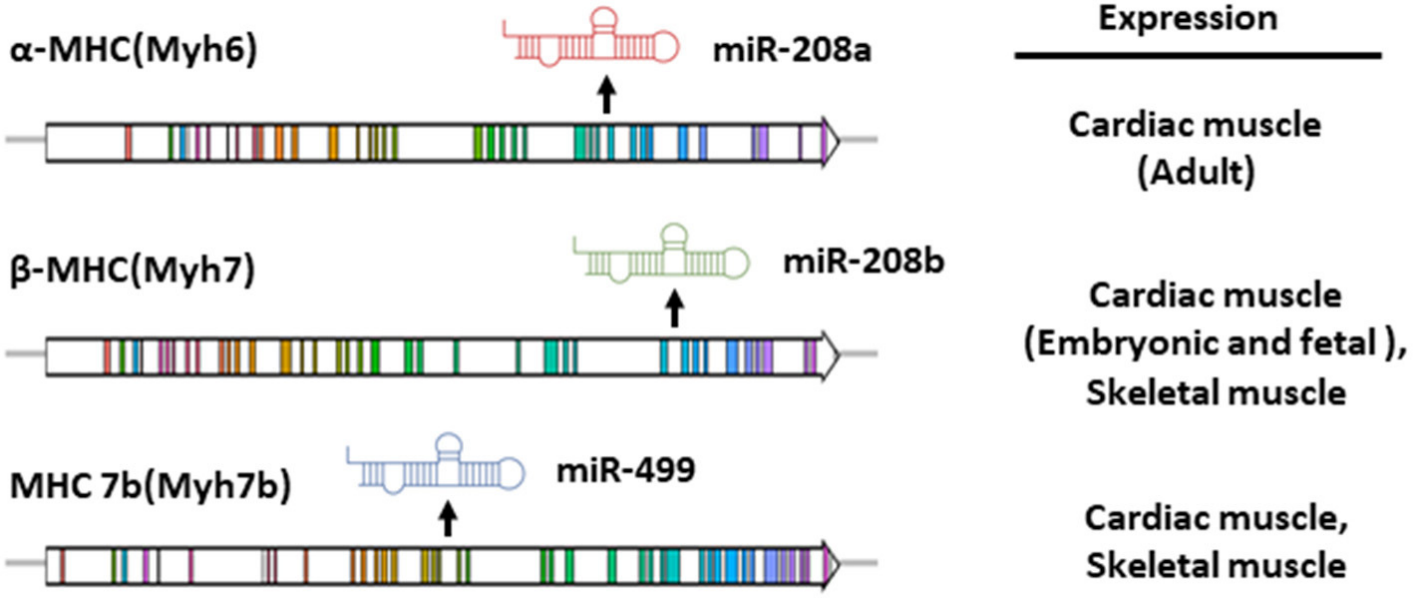
Gene structure and expression pattern of miR-208a, miR-208b and miR-499 and their host genes in mouse. The intronic miR-208a, miR-208b and miR-499 are co-transcribed with their host genes, Myh6, Myh7 and Myh7b respectively.

MiR-208a belongs to the miRNA family, which also includes miR-208b and miR-499 (21, 23). Members of this family contain nearly identical seed sequences, thus can target certain common downstream mRNAs and may be functionally redundant (21). These three miRNA are encoded by the intron regions of Myh6, Myh7, and Myh7b genes, respectively (Figure 1) (21, 23). These three genes encode myosin proteins involved in multiple muscle pathophysiological processes. In mouse, the Myh7 gene encodes the β-isoform of myosin heavy chain (β-MHC) and is highly expressed in embryonic or neonatal cardiomyocytes, while Myh6 encodes the α-isoform of myosin heavy chain (α-MHC), the predominant myosin heavy chain (MHC) isoform in adult heart (24, 25). Cardiac remodeling is usually accompanied by myofibrillar remodeling, a shift in MHC isoform content from α(adult) to β(fetal) in cardiac muscle. Such α-MHC to β-MHC switch may be a maladaptive response and can accelerate the pathogenic remodeling (26–28).

The intronic miR-208a, miR-208b and miR-499 are co-transcribed with their host genes. Spatially, miR-208b and miR499 are expressed in both skeletal and cardiac muscle tissues, while miR-208a is specifically presented in the heart (20, 21, 23). In parallel with the expression of α-MHC and β-MHC in the heart which is developmentally regulated, cardiac miR-208b is mainly expressed in the embryonic or neonatal stages, while miR-208a is enriched in the adult cardiac muscle (21, 23). Notably, humans display an entirely distinct myosin expression pattern. MHY7 is the major left ventricular MHC in the adults, whereas *MYH6* eocodes the myosin enriched in developing human ventricle and adult atrium (29). Owing to the expression pattern of their host genes, expression of miR-208 family members in adult human hearts showed prominent chamber specificity. MiR-208a is abundant in atrial myocardium, while miR-208b is preferentially expressed in left ventricles (30).

Expression of miR-208a in the heart is also regulated at the posttranscription level. A double-strand RNA binding protein TRBP is required for the normal posttranscriptional processing of miR-208a. TRBP functions as co-factor of DICER and may confer the dicing specificity or preference for the cleavage of pre-miRNAs (31–33). Genetic abrogation of TRBP in the heart led to the dysregulation of a small subset of miRNAs, among which, miR-208a, miR-208b and miR-499 were the significantly and substantially downregulated ones. The previous study indicates that these three myomiRs appear to be the primary targets of TRBP (34). It is not clear how precursors of these miRNAs are recognized by TRBP machinery or how TRBP “selectively” regulates processing of pre-miR-208a, pre-miR-208b and pre-miR-499 (and several other pre-miRNAs) in the heart. Since TRBP is a double-strand RNA binding protein, the stem regions of pre-miR-208a, pre-miR-208b and pre-miR-499, which have certain sequence similarity, may act as the *cis*-elements mediating the recognition. Intriguingly, abrogation of TRBP in the skeletal muscle did not alter the level of miR-499 or its target Sox6 (34, 35). This observation indicates that regulation of miR-499 (and likely also miR-208a) processing by TRBP is context-dependent. One possibility is that additional cardiac-specific cofactors may exist, act in *trans* and participate in reshaping such specificity or preference. It is unclear whether the posttranscriptional regulatory events are conserved in humans. Further investigation on the underlying mechanisms will offer important clues for understanding of the specificity of miRNA post-transcriptional processing.

Intriguingly, expression of miR-208 exhibits certain gender differences. The level of miR-208 in female is 16 and 21-fold higher than that in male at 15 and 21 weeks of age, displaying a female-biased pattern (36). In addition, in both Zucker Lean and in Zucker diabetic fatty rats, which exhibit cardiac hypertrophy, the expression of cardiac miR-208a is much higher in female compared to male, further demonstrating the gender differences (37). The mechanisms need to be addressed in the future.

## The Functional Roles of miR-208a

**Figure 2:**
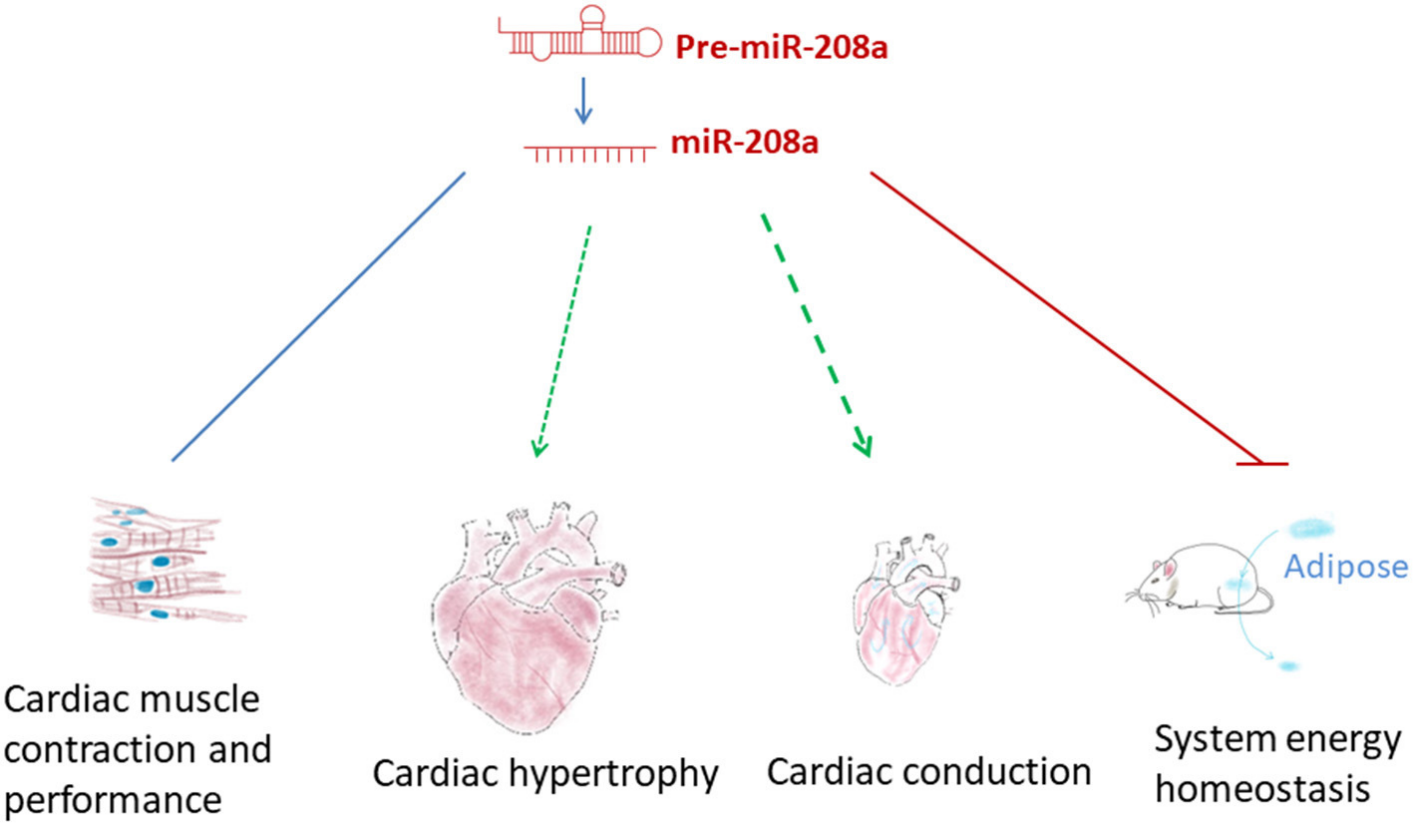
The functional roles of miR-208a. miR-208a participates in regulating multiple pathophysiological processes, such as cardiac hypertrophy, disordered cardiac conduction and contraction and system energy dyshomeostasis.

**Figure 3:**
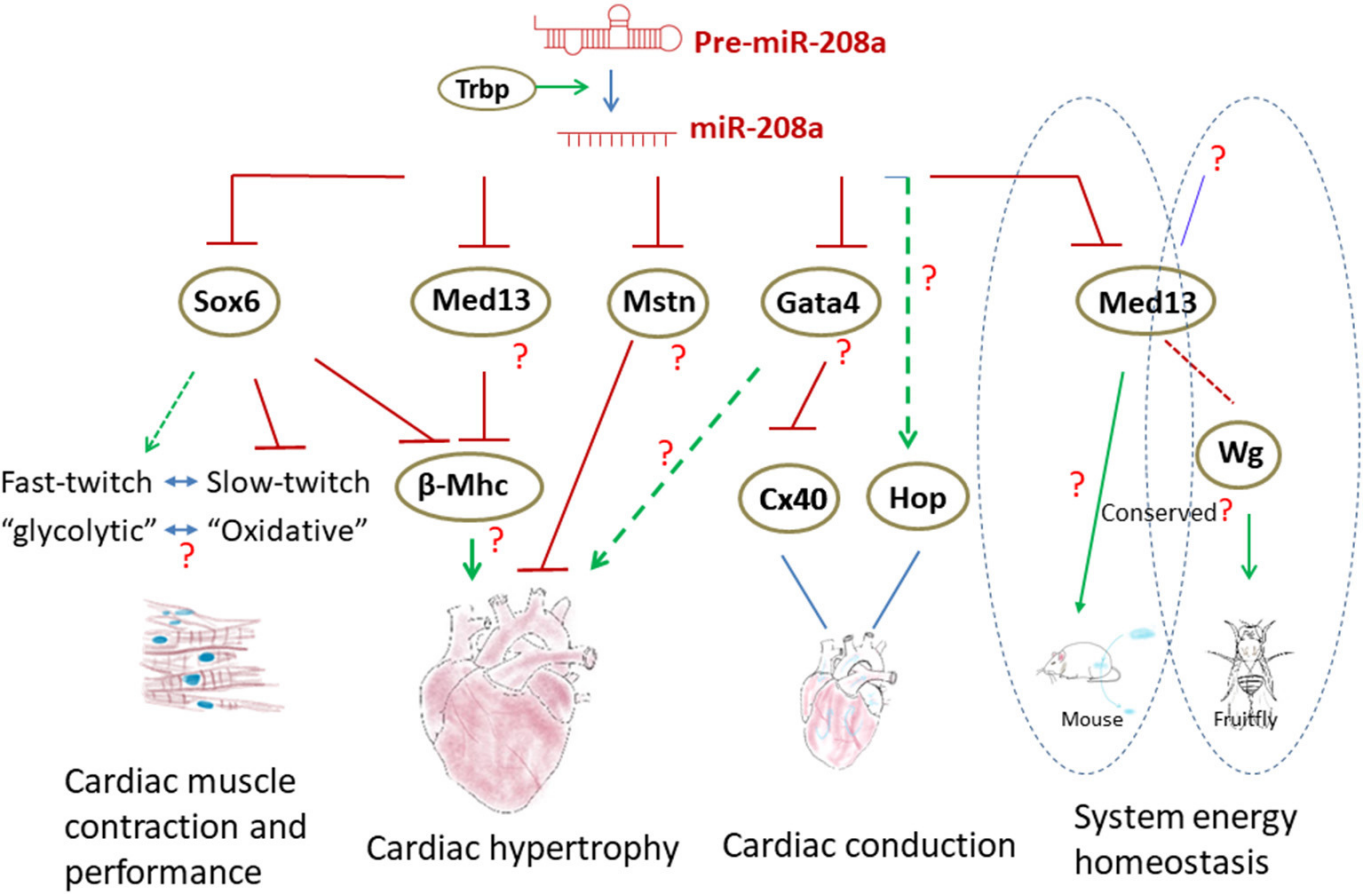
The regulatory networks involving miR-208a in the heart. Normal biogenesis of miR-208a in the heart requires TRBP. miR-208a regulates cardiac function by modulating the expression of downstream genes. In addition, miR-208a in the heart can also control the system energy homeostasis through MED13. Wg was found to be a downstream factor of MED13 in *Drosophila* to regulate metabolism, yet it is not clear if the processes are conserved in mammals.

Abnormal expression of miR-208a has been observed in the onset of diseases such as cardiac hypertrophy and heart failure, suggesting that this cardiac specific miRNA may participate in modulating heart function (38–41). Indeed, miR-208a has been found to be involved in a broad array of cellular processes in cardiac pathogenesis by targeting a wide range of downstream mRNAs (Figures 2, 3). Yet, its functional roles and effects in the heart are more complicated than expected, and many questions remain regarding the underlying mechanisms.

### miR-208a and Cardiac Hypertrophy

Although it is one of the most abundant miRNAs in adult cardiomyocytes, when miR-208a was deleted in mouse (*miR*–208*a*^−/−^ or miR-208a KO), no obvious cardiac phenotype, but only a slight reduction in fractional shortening, was seen (20). Abrogation of miR-208a only resulted in very mild phenotypes in the heart at the basal level, whereas it had more profound functional influences in stress models (20, 23). In response to pressure overload or activation of calcineurin signal, miR-208a knockout mouse exhibited resistance to cardiac remodeling, showing virtually no hypertrophic growth of cardiomyocytes or fibrosis (20). Thus, miR-208a is required for stress-induced cardiac pathogenesis. The pro-hypertrophic effect of miR-208a was further demonstrated in the gain-of-function studies. Overexpression of miR-208a in mouse heart is sufficient to induce cardiac hypertrophy, which is evidenced by increased ventricle wall thickness and cross-sectional cell area of cardiomyocytes, and enlarged ventricle chambers (23). At the molecular level, inactivation of miR-208a broadly altered gene expression in mouse hearts. In particular, the transcripts of genes encoding early response factors, heat shock proteins, and skeletal muscle fast-twitch myofiber proteins were substantially upregulated in miR-208a mutant hearts (20). However, the pathophysiological relevance of the altered gene expression pattern is not clear, and the mechanisms by which miR-208a regulates cardiac hypertrophy are still elusive.

There are several topics with regard to miR-208a and cardiac hypertrophy yet to be resolved. Expression of β-MHC in adult cardiac muscle not only is one of the hallmarks of hypertrophy, but appears to be a maladaptive response in the heart, since it can accelerate the pathogenic remodeling (26–28). In miR-208a mutants, cardiac stress failed to upregulate β-MHC, while miR-208a transgene in mouse heart can potently induce the expression of β-MHC and lead to hypertrophy (20, 23). Intriguingly, elevated expression of miR-208a has been implicated in diabetic cardiomyopathy in human patients. The functional consequences were linked to the up-regulation of β-MHC and the α/β-MHC switch, too (42). These findings raise the question of whether the pro-hypertrophic/pro-remodeling function of miR-208a is attributed to its regulatory effects on β-MHC. If yes, how does miR-208a regulate β-MHC? Previous studies have suggested that it could be mediated by numerous transcription regulators, including Med13 (or thyroid hormone–associated protein 1, Thrap1) and Sox6, the transcripts of which contain the binding sites and may be the direct targets of miR-208a (20, 23, 34). The amount of Sox6 transcripts was increased in miR-208 null mice, thus,miR-208a can inhibit Sox6 at the mRNA level in the heart (21, 34). Overexpression of Sox6 in heart did result in the downregulation of β-MHC, which was also observed in *miR*–208*a*^−/−^ mice (21, 43, 44). However, in miR-208a transgenic mice exhibiting upregulation of β-MHC (23), the mRNA level of Sox6 was unaltered (34), indicating additional unrecognized molecular events may be involved. Med13 (Thrap1) is another target and has been thought to function downstream of miR-208a to regulate of myosin expression. Nevertheless, as shown in a study by Grueter et al. (45), transgenic expression of Med13 resulted in upregulation of β-MHC, which appears contradictory to the observation in *miR*–208*a*^−/−^ mice. In addition, no hypertrophy was detected when Med13 gene was deleted in cardiac muscle (45). Thus, the roles of miR-208a/Sox6 and miR-208a/Med13 axes in modulating β-MHC expression and hypertrophy in the heart appear obscure.

Notably, the induced expression of β-MHC *in vivo* by miR-208a transgene was heterogenous in the myocardium (23). It may be caused by the “mosaicism” of transgene expression. It is also likely due to the heterogeneity of the cardiomyocytes in the heart, that individual ones may differentially respond to miR-208a overexpression (23). In addition, β-MHC upregulation in the heart may not be a primary effect, but secondary to the cardiac abnormalities induced by miR-208a transgene (46). The intra-and inter-cellular molecular events underlying these observations need to be further investigated in the future.

Although can accelerate the pathogenesis in the heart, elevated expression of β-MHC alone is not sufficient to induce cardiac remodeling. Thus, it is still unclear how and how much the upregulated β-MHC expression is relevant to cardiac hypertrophy in miR-208a transgenic (miR-208TG) mouse. Is it a correlation or the causality? As shown in the study by Callis, there was no association between the state of β-MHC induction and hypertrophic growth of individual cardiomyocytes, suggesting that upregulation of β-MHC may be not an obligate component of miR-208a-induced hypertrophy (23). Then, what else can participate in mediating the pro-hypertrophic effects of miR-208a? Cardiac hormone atrial natriuretic factor (ANF) is another molecular hallmark of cardiac remodeling (4). However, no significant change of ANF mRNA abundance was detected in miR-208TG hearts (23). Levels of miRNA markers, including miR-1, miR-133 and miR-29a (downregulated in hypertrophy), miR-125b (upregulated in hypertrophy) were not altered either (23). These observations indicate that overexpression miR-208a may induce hypertrophic growth of cardiomyocytes without affecting the aspects of these known pathways.

MiR-208a has been shown to target *Myostatin(Mstn)* (23), which encodes a negative regulator of muscle growth (47). Genetic inactivation of Mstn signaling in the adult murine heart caused cardiac hypertrophy, which phenotypically resembled the consequences of miR-208a overexpression (23, 48). This finding indicates that *Mstn* may be *one* of the downstream targets mediating the pro-hypertrophic function of miR-208a. Yet, it is necessary to experimentally validate the role of Mstn particularly in miR-208a-induced hypertrophy and further confirm the regulatory effects of miR-208a/*Mstn* axis on cardiac remodeling.

One thing, which makes it challenging to decipher the mechanisms of miR-208a, is that this versatile miRNA can target not only anti-hypertrophic genes, but also those encoding pro-hypertrophic factors. For instance, expression of Gata4 in the heart, a transcription factor required for cardiac hypertrophy, was found to be inhibited by miR-208a (23). It is unclear what the role(s) of Gata4 in miR-208a-induced cardiac hypertrophy is. How does miR-208a exert its pro-hypertrophic effects when the downstream targets with opposing functions (anti-hypertrophic Mstn vs. pro-hypertrophic Gata4) co-present in the heart? It may be dependent on miR-208a abundance, the availability of the different targets and the physiological contexts. Comprehensive and systemic characterization of the “miR-208a network” and a deeper understanding of miR-208a activity in the different relevant physiological and pathological contexts are needed, in order to ultimately define the functions of this important miRNA in cardiac hypertrophy. The in-depth further investigation on miR-208a will also offer important insights into the regulatory mechanisms of cardiac remodeling (Figure 3).

### miR-208a and Cardiac Conduction

miR-208a is also a key regulator of cardiac conduction. Loss of miR-208a resulted in cardiac conduction abnormalities (23). Expression of transcription factor homeodomain-only protein (Hop) and gap junction protein connexin 40 (Cx40) was dramatically down-regulated in the hearts of miR-208a null mice, suggesting that miR-208a is required for the normal expression of these cardiac conduction-related genes (23). MiR-208a may indirectly regulate Hop and Cx40 by targeting GATA4 (23). However, loss of miR-208a only slightly increased the level of GATA4 (23), which appears unlikely to be sufficient to cause such dramatic alteration of Hop and Cx40 expression. Thus, additional unidentified miR-208a downstream targets and other factors may participate in and contribute to the process. The miR-208a transgenic mice exhibited cardiac conduction defects, too (23). Yet, apparently, the abnormalities were not concurrent with the dysregulation of Hop and Cx40. As shown in the study by Callis, overexpression of miR-208a did not alter the expression of Cx40 (23). Growing evidences have suggested that the targeting specificity and capacity of miRNAs are dose-dependent and are often sensitive to the biological contexts (49). Although it is unknown whether the expression of Gata4 was downregulated in miR-208aTG hearts, the cardiac conduction abnormalities observed in miR-208a gain- and loss-of function backgrounds may be attributed to different regulatory pathways and mediated by different downstream targets (Figure 3).

### miR-208a and Slow-/Fact- Twitch Contractile Gene Expression

The primary function of the heart is to circulate blood by beating and contracting. Defects in contraction often result in severe cardiac disorders. The contractile apparatus in myocytes consists of sarcomeric proteins, which can be broadly classified into 2 major types: fast-twitch and slow-twitch (24, 50). The major type of contractile proteins in cardiomyocytes is slow-twitch, and fast-twitch genes are expressed at much lower levels in the heart (24, 25, 50, 51). However, it has not been well documented how the pattern of fast-/ slow-twitch gene expression is established in cardiomyocytes, and the pathophysiological relevance is not fully understood. As shown in the study by Ding et al., heart-specific inactivation of Trbp (*Trbp*^cKO^) in mice resulted in progressive cardiac remodeling, concurrent with a “slow-to-fast” shift in myofiber gene expression in the heart, downregulation of normal cardiac slow-twitch myofiber genes and increased expression of genes encoding fast-twitch contractile proteins (34). Dramatic downregulation of miR-208a was observed in *Trbp*^cKO^ hearts and appeared to be responsible for the cardiac defects. Indeed, reintroduction of miR-208a into Trbp mutant hearts substantially corrected the fast- and slow- twitch myofiber gene expression pattern and rescued the cardiac abnormalities (34). These findings not only implied that the unbalanced fast-/ slow-twitch gene expression and the possible desynchronized myofilament activation could be the cause of the cardiac defects, but also demonstrated the crucial role of miR-208a in establishing or maintaining the proper expression pattern of slow-/ fast-twitch myofiber genes in the heart.

The regulatory effects of miR-208a on slow-/fast- twitch contractile gene expression in the heart appears to be mediated mainly by its downstream target Sox6 (34). Sox6 has been shown to modulate the expression of slow-/fast- twitch myofiber genes in skeletal muscle (43, 44, 52, 53). The similar regulatory events also occur in cardiac muscle. Overexpression of Sox6 in cardiac muscle resulted in the “slow-to-fast” shift in myofiber gene expression, recapitulating the effects of Trbp inactivation (34). Intriguingly, the regulatory effect of Trbp on Sox6 and contractile gene expression appeared to be quantitively correlated with the postnatal ages. It was subtle in neonatal cardiac muscle in which miR-208b is more abundant, but more substantial at adult stage when miR-208a is predominant in the heart (34). Similar to miR-208a, miR-208b is regulated by Trbp, too. Sox6 can also be targeted by miR-208b (21), which has an identical seed sequence to miR-208a, only differing at 3 nucleotides in the 3' region. What is the observed “age-dependence” accounted for, then? It is likely that the targeting capacity of miR-208b on Sox6 is not as strong as that of miR-208a, due to the dissimilarity in sequence outside of the seed region. Or, it is possible that the effects are context-/stage-dependent, and the regulatory axis is effective preferentially in adult hearts.

Skeletal muscle consists of two major types of myofibers, that type I myofibers mainly expressing slow-twitch contractile genes produce relatively less force but can sustain long-lasting contractions, and type II fibers which express fast-twitch contractile genes can support high-intensity and short-duration contractions (51). The molecular motors in myocytes require energy to sustain the contraction. These two types of myofibers have different energy demands and thus preferentially utilize different metabolic pathways to generate ATP. The fast -twitch (type II) myofibers are usually “glycolytic” (anaerobic), whereas the slow ones (type I) are “oxidative” (aerobic) (50, 51, 54). The energetic states are correlated with the myofiber types. Although cardiomyocytes, unlike skeletal muscle cells, appear not to undergo fast- and slow-twitch fiber-type speciation, the findings from the study by Ding et al. (34), raised a question of whether the “slow-to-fast” shift in myofiber gene expression in Trbp mutant hearts is accompanied with a change(s) in metabolic pathways (fatty acid oxidation to glycolysis) (55). It will be interesting to study the link or coupling between these two events in cardiac muscle and the involvement of miR-208a in the processes in the future (Figure 3).

### miR-208a and Systemic Energy Homeostasis

miR-208a not only regulates heart function, but also participates in modulating systemic energy homeostasis. As shown in the study by Grueter et al. (45), mice administered with miR-208a inhibitor were resistant to high-fat diet-induced obesity, and exhibited improved insulin sensitivity as well as glucose tolerance. Transgenic expression of Med13, a target of miR-208a, in cardiac muscle attenuated the metabolic defects in mouse models of obesity, phenocopying the effects of miR-208a inhibition. The study demonstrated that miR-208a/Med13 axis in cardiac muscle was involved in regulating the energy homeostasis in distant organs, including fat tissue and liver, and indicated that miR-208a could be a promising therapeutic target for metabolic disorders such as type 2 diabetes and obesity (Figures 2, 3). In addition, rapamycin and nebivolol were reported to inhibit weight gain in rodent model of obesity and in human patients (56–59). The effects are similar to the effect of miR-208a inhibition. A further study indicates that both rapamycin and nebivolol may suppresses the up-regulation of miR-208a via inhibiting mTORC1 activation, thus increasing the level of MED13 and conferring the resistance of obesity (60).

The heart is more than a “pump” and may act as an endocrine organ to regulate the energy storage or dissipation of the whole body (45, 61). A tantalizing question is, what mediates endocrine function of the heart. Is there a “slimming” factor(s) produced in cardiomyocytes, regulated by the miR-208a/Med13 axis, and secreted into the bloodstream, so that it modulates the whole-body metabolism? This hypothetical answer can be tested with the parabiosis model, which allows exchange of whole blood between 2 animals (62). It is also possible that the signal output downstream of miR-208a/Med13 axis in cardiac muscle may be initially delivered to the brain as a relay system to other tissue and organs (61).

Intriguingly, Med13 has been found to inhibit lipid accumulation in *Drosophila* (63), thus the anti-obesity function appears to be conserved in invertebrates. Taking advantages of *Drosophila*, which is a good *in vivo* model for genetic analysis of obesity and an ideal system for studying inter-organ crosstalk, Lee et al. (63). Identified the Wingless(Wg) peptide as a circulating “slimming” factor downstream of Med13 and released by cardiac and skeletal muscle. Although autonomous activation of Wnt signaling in adipose can reduce fat mass in mice (64), it is not clear whether the Wnt ligand(s), in particular Wnt1, the mammalian ortholog of Wg, is regulated by miR-208a/Med13 axis in the heart and mediates the cross-organ communications, as well (Figure 3).

## miR-208a as a Biomarker

Though exclusively expressed in cardiac muscle, miR-208a can be secreted by cardiomyocytes into serum and plasma, in response to cardiac stresses. The abundance of circulating miR-208a was found to be altered concurrently with cardiac pathogenesis in numerous studies (65–67). For instance, in mouse models treated with isoproterenol, plasma level of miR-208a was closely correlated to circulating cardiac troponin I, which is a widely used biomarker for myocardial injury (65, 68). Raised level miR-208a in plasma was also detected in human patients with myocardial injury (66). Although it is unclear whether the upregulation of circulating miR-208a is adaptive or maladaptive and whether it has any functional consequence(s) in cardiac remodeling, growing evidences suggested that it could be a promising, non-invasive diagnostic biomarker for cardiac defects.

## miR-208a as a Therapeutic Target

Animals with genetic deletion of miR-208a, at the basal level, appeared to be phenotypically normal or only have very mild phenotypes, while exhibited resistance to cardiac remodeling in response to stresses (20). Inhibition of miR-208a by subcutaneous delivery of the locked nucleic acid-modified antisense oligonucleotides (69–71) (antimiR-208a) could dose-dependently blunts stress-induced cardiac pathogenesis in a rat model of diastolic heart failure (Dahl salt-sensitive rats) (72). The antimiR-208a significantly improved the cardiac function, overall health and survival of the animals, and no adverse side effects were detected on the treatment (72). These findings indicated that targeting miR-208a could be an efficient and a safe way to prevent cardiac remodeling in the heart with minimal side effects on normal tissues.

Altered expression of miR-208 family has been implicated in the onset of cardiac hypertrophy and heart failure in human patients (38–41), indicating that this miRNA and its family members (miR-208b, miR-499) hold great potential as the therapeutic targets. Although their expression patterns in humans are different from those in rodents, the studies using mouse and rat models have been offering important insights and clinical implications. miRNAs exert their functions by regulating their downstream targets, most of which are protein-coding genes. For many identified miRNA:mRNA pairs, the sequence complementation is well conserved in both primates and rodents. Yet, owing to the difference of α/β MHC (their host genes) expression between mice and humans, investigations using non-human primate animal models may be needed to further validate the therapeutic effects prior to full clinical application.

## Perspective

MiR-208a is one of the cardiac miRNAs which relatively have been well investigated. Yet, as discussed above, our understanding of this miRNA appears still rudimentary. It may be not just for miR-208a. According to Dr. Eric Olson, “there has been a tendency to oversimplify the mechanistic basis of miRNA functions…” (73). Numerous topics need to be addressed. For miR-208, the biological functions and the mechanisms of this miRNA, its multiplicity, the broad range of downstream targets and the complex regulatory network(s), remain very elusive. This miRNA holds great potential as a therapeutic target, however, extensive studies and analyses are required prior to full clinical application.

Safety is always an important issue for therapeutics. Despite the promise of miR-208a-based therapeutics, it is necessary to evaluate the long-term effects of antimiR-208a or other miR-208a inhibitors in various settings. There is a long way to go, but further investigations on this unique miRNA will contribute to the development of new therapeutic approaches to treat heart diseases.

## Author Contributions

X-HH, J-LL, and JD conceived the presented idea and prepared the manuscript. X-HH, J-LL, X-YL, S-XW, Z-HJ, and S-QL summarized the literature and produced the figures. X-YL, S-XW, Z-HJ, S-QL, and JL reviewed and edited the manuscript. X-HH, J-LL, JL, and JD drafted the final version of the manuscript. All authors contributed to the article and approved the submitted version.

## Funding

The work was supported by National Natural Science Foundation of China (31771371 and 31970654) and Natural Science Foundation of Shaanxi Province for Fundamental Research (2019JM-058).

## Conflict of Interest

The authors declare that the research was conducted in the absence of any commercial or financial relationships that could be construed as a potential conflict of interest.

## Publisher's Note

All claims expressed in this article are solely those of the authors and do not necessarily represent those of their affiliated organizations, or those of the publisher, the editors and the reviewers. Any product that may be evaluated in this article, or claim that may be made by its manufacturer, is not guaranteed or endorsed by the publisher.
